# Genetic and Clinical Characteristics of Patients With Tumor Mutation Burden‐High Unresectable Pancreatic Cancer and the Efficacy of Pembrolizumab Treatment

**DOI:** 10.1002/cnr2.70492

**Published:** 2026-02-11

**Authors:** Yugo Kai, Kenji Ikezawa, Kazuhiro Kozumi, Makiko Urabe, Ryoji Takada, Takumi Kinomoto, Takanori Masumoto, Masaki Kawabata, Hiroki Kishimoto, Kana Hosokawa, Kaori Mukai, Tasuku Nakabori, Yuko Yamaguchi, Naotoshi Sugimoto, Kazuyoshi Ohkawa

**Affiliations:** ^1^ Department of Hepatobiliary and Pancreatic Oncology Osaka International Cancer Institute Osaka Japan; ^2^ Department of Gastroenterology and Hepatology Kansai Rosai Hospital Amagasaki Japan; ^3^ Department of Genetic Oncology Osaka International Cancer Institute Osaka Japan

**Keywords:** comprehensive genomic profiling test, pancreatic cancer, pembrolizumab, tumor mutation burden‐high

## Abstract

**Background:**

Pembrolizumab is approved for treating patients with advanced solid tumors exhibiting high tumor mutation burden (TMB), including pancreatic cancer. However, owing to the rarity of TMB‐high pancreatic cancer, its genetic and clinical characteristics, alongside the therapeutic effectiveness of pembrolizumab, remain unclear.

**Aims:**

To investigate the characteristics and assess the effectiveness of pembrolizumab in this patient population.

**Methods and Results:**

We retrospectively reviewed data of 293 patients with unresectable or recurrent pancreatic cancer who underwent comprehensive genomic profiling at our hospital between December 2019 and April 2023. TMB‐high was observed in 13 cases (4.4%), including four patients with microsatellite instability‐high (MSI‐H) (1.4%). Two patients exhibited germline *BRCA2* mutations: one with adenocarcinoma and the other with acinar cell carcinoma. Germline mutations in *MLH1* and *MSH6* were each identified in one case, both exhibiting MSI‐H plus TMB‐high. The frequency of pathogenic mutations in *KRAS*, *TP53*, *CDKN2A*, and *SMAD4* was notably high. *KRAS* mutations were detected in 12 of the 13 patients (92.3%). Pembrolizumab was administered to six patients, yielding an objective response rate of 33.3% and a disease control rate of 66.7%. Among the three patients with MSI‐H plus TMB‐high, two achieved partial response, and the median progression‐free survival for all three patients was 227 days. Among the three microsatellite stable (MSS) plus TMB‐high cases, two exhibited stable disease, and the median progression‐free survival for all three patients was 90 days.

**Conclusion:**

The frequency of TMB‐high was 4.4%, which is slightly higher than that previously reported. Pembrolizumab demonstrated greater efficacy in patients with MSI‐H plus TMB‐high while also exhibiting some efficacy in patients with MSS plus TMB‐high.

## 1 Introduction

Pancreatic cancer is associated with the poorest prognosis among malignancies [1]. In the United States, recent statistics estimate approximately 66 440 new cases and 51 750 mortalities annually, with both numbers showing an upward trend [2]. The 5‐year survival rate is approximately 13% [2]. In Japan, as in other countries, cytotoxic chemotherapy remains the standard treatment for unresectable pancreatic cancer [3]. First‐line chemotherapy typically includes combination regimens such as FOLFIRINOX or gemcitabine plus nab‐paclitaxel (GnP) [3, 4, 5]. Recently, the randomized phase 3 NAPOLI‐3 trial demonstrated that NALIRIFOX is superior to GnP in the treatment of metastatic pancreatic cancer [6]. For second‐line treatment, the global phase 3 NAPOLI‐1 trial shows a survival benefit with liposomal irinotecan combined with 5‐fluorouracil/leucovorin (nal‐IRI + 5‐FU/LV) after gemcitabine‐based therapy [7]. The clinical efficacy of nal‐IRI + 5‐FU/LV has also been reported in Japan [8, 9]. Despite gradual improvements in chemotherapy for pancreatic cancer, treatment outcomes remain unsatisfactory, necessitating the development of more effective treatments, including immunotherapy and precision medicine based on genomic profiling.

Tumor mutation burden (TMB) is the total number of somatic mutations within the tumor genome [10]. The TMB score is quantified as mutations per megabase (mut/Mb) [11]. TMB‐high solid tumors generate more neoantigens, which induce immune responses and increase the likelihood of tumor recognition by the host immune system [12, 13]. Pembrolizumab, a humanized monoclonal antibody targeting the programmed cell death 1 (PD‐1) protein, activates the host immune response against tumors [14]. Clinical trials, including KEYNOTE‐158, demonstrate the efficacy of pembrolizumab in non‐colorectal solid tumors with microsatellite instability‐high (MSI‐H) or mismatch repair deficiency (dMMR) [15, 16]. In a separate cohort of the KEYNOTE‐158 trial, Pembrolizumab treatment yielded an objective response rate of 29% in 102 patients with TMB‐high (TMB ≥ 10 mut/Mb) tumors [17]. Based on these findings, pembrolizumab has been approved for treating advanced solid tumors with MSI‐H or TMB‐high status [18, 19]. However, the KEYNOTE‐158 trial did not include patients with pancreatic cancer [17]. Owing to the low prevalence of TMB‐high pancreatic cancer, the genomic characteristics of this subset and the therapeutic effects of immune checkpoint inhibitors (ICIs), including pembrolizumab, remain unclear. Quintanilha et al. report a 1.3% frequency of TMB‐high pancreatic ductal adenocarcinoma (PDAC) based on comprehensive genomic profiling (CGP), along with favorable median overall survival among patients treated with ICIs. However, the study used a clinical genome database but lacked detail on individual patient conditions or ICI treatment courses [20]. Similarly, Sakaida et al. report the frequency and genetic characteristics of TMB‐high pancreatic cancer in Japan, but this study also used the Japanese Center for Cancer Genomics and Advanced Therapeutics (C‐CAT) CGP assay database. Nonetheless, data on the efficacy of pembrolizumab treatment were not provided [21]. Therefore, in this single‐center retrospective study, we aimed to analyze the clinical and genetic characteristics of TMB‐high pancreatic cancer using the CGP assay and examine the efficacy of pembrolizumab in affected patients.

## 2 Methods

### 2.1 Study Design and Patient Selection

We retrospectively reviewed the clinical data of 293 patients with unresectable or postoperatively recurrent pancreatic cancer who underwent CGP testing between December 2019 and April 2023 at a Japanese cancer referral center (Osaka International Cancer Institute). All pancreatic cancer cases in this study were pathologically confirmed, and all histological types, including special subtypes, such as anaplastic carcinoma and intraductal papillary mucinous adenocarcinoma, were eligible. No exclusion criteria based on patient characteristics were applied; for example, patients with concurrent malignancies or those who had received specific anticancer therapies were not excluded. However, cases in which CGP testing was planned but later discontinued because the sample was deemed unanalyzable were excluded. Clinical data were obtained from medical records, and follow‐up data were censored on April 30, 2024.

### 2.2 Genomic Profiling Tests

The CGP tests included the OncoGuide NCC Oncopanel System (Sysmex Corporation, Kobe, Japan), FoundationOne CDx (F1CDx) and FoundationOne Liquid CDx (F1L) (Foundation Medicine, Cambridge, MA, USA). The test type was selected based on the clinical judgment of the attending physician in consultation with the patient. For tissue‐based CGP assays, all CGP tests were performed using formalin‐fixed paraffin‐embedded specimens, which were deemed appropriate by the attending physician and pathologist. When an appropriate tissue sample was not available or could not be freshly collected, CGP testing was performed using liquid biopsy.

### 2.3 Sample Collection and Review Process

Tissue sampling methods were determined by the attending physicians. All cases were reviewed by expert panels at molecular tumor board meetings comprising oncologists, geneticists, pathologists, bioinformaticians, and genetic counselors [22]. The board evaluated reported variants for clinical significance and determined their somatic or germline origin by integrating genomic findings with clinical information. TMB was classified as high (≥ 10 mut/Mb), irrespective of the CGP assays, by an expert panel in our hospital.

### 2.4 Statistical Analysis

Categorical and continuous variables were compared using Fisher's exact test and the Mann–Whitney *U* test, respectively. Progression‐free survival (PFS) during pembrolizumab treatment was compared using the Kaplan–Meier survival curves and log‐rank test. A two‐sided *p* < 0.05 was considered statistically significant. Statistical analyses were performed using the EZR software version 1.54 (Saitama Medical Center, Jichi Medical University, Saitama, Japan).

### 2.5 Ethics Statement

This study was approved by the Institutional Review Board of the Osaka International Cancer Institute (20148‐8) and was performed in accordance with the Declaration of Helsinki. The requirement for written informed consent was waived through an opt‐out process on our website. This waiver was granted by the Institutional Review Board of Osaka International Cancer Institute.

## 3 Results

### 3.1 Patient Characteristics and Prevalence of Patients With TMB‐High and MSI‐H Pancreatic Cancer in CGP Assays

**FIGURE 1:**
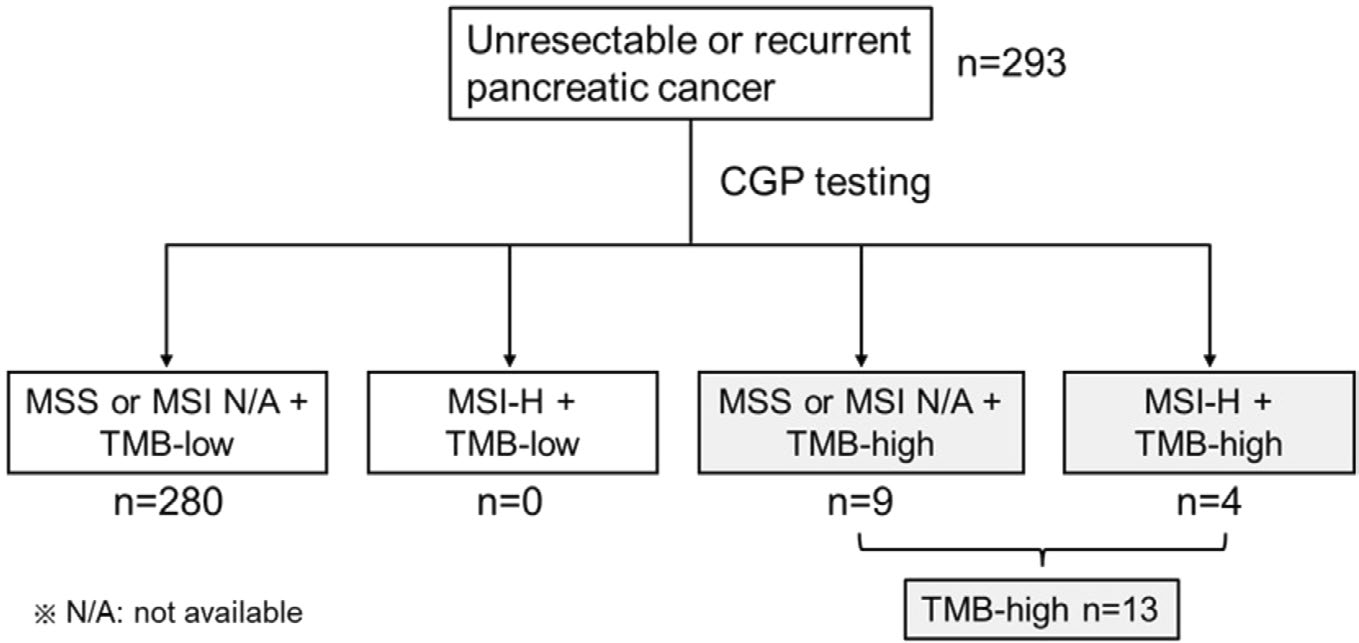
Distribution of tumor mutation burden (TMB)‐high and microsatellite instability‐high (MSI‐H) status in patients with pancreatic cancer.

Overall, 293 patients with unresectable pancreatic cancer who underwent CGP testing were analyzed. Table 1 shows the baseline characteristics of the patients. Of these, 162 patients (55.3%) were males, with a median age of 64 years (range: 37–85 years). Most patients had adenocarcinomas (94.2%), whereas 2.7% had adenosquamous carcinomas (Asc) and 1.4% had acinar cell carcinomas (ACC). The CGP assays used in this study included F1CDx in 194 patients (66.2%), NCC in 60 patients (20.5%), and F1L in 39 patients (13.3%). Table 1 also shows the cancer tissue sampling methods and organs used for CGP testing. When comparing the TMB‐low and TMB‐high groups, no differences were observed in sex, histological classification, or CGP assay. The median age was higher in the TMB‐high group than in the TMB‐low group. Regarding the sampling method (excluding liquid biopsy), all cases in the TMB‐high group were biopsy specimens, with no surgical specimens included. Furthermore, pancreatic specimens accounted for 11 cases (91.7%) in the TMB‐high group, representing a significant difference in the type of sampling organ compared with the TMB‐low group (*p* = 0.021). Table 2 shows the TMB and MSI status from the CGP examination. The success rates for the TMB and MSI assessments were 93.9% and 84.0%, respectively. TMB scores could not be determined (CBD) in 18 cases (6.1%) owing to low sequencing coverage or low tumor purity. MSI status was unavailable in 21 cases (7.2%) because the NCC Oncopanel did not initially support MSI analysis, and could not be determined in 26 cases (8.9%) owing to low sequencing coverage or low tumor purity. Among the 293 patients who underwent CGP testing, 13 cases (4.4%) had TMB‐high tumors. Stratified by CGP assays, TMB‐high rates were as follows: nine out of 194 (4.6%) in F1CDx, three out of 60 (5.0%) in NCC, and one out of 39 (2.6%) in F1L. Similarly, MSI‐H was detected in four patients (1.4%), all exhibiting TMB‐high (Figure 1).

**TABLE 1:** Patient characteristics.

	All patients	TMB‐low group	TMB‐high group	*p*
Number of patients, *n*	293	280	13	—
Sex				
Male, *n* (%)	161 (54.9%)	125 (44.6%)	6 (46.2%)	1.000
Female, *n* (%)	132 (45.1%)	155 (55.4%)	7 (53.8%)	
Median age, years (range)	64 (37–85)	64 (37–85)	71 (50–75)	0.026
Histological classification				
Adenocarcinoma, *n* (%)	276 (94.2%)	265 (94.6%)	11 (84.6%)	0.129
Adenosquamous carcinoma, *n* (%)	8 (2.7%)	7 (2.5%)	1 (7.7%)	
Acinar cell carcinoma, *n* (%)	4 (1.4%)	3 (1.1%)	1 (7.7%)	
Others, *n* (%)	5 (1.7%)	5 (1.8%)	0 (0.0%)	
CGP assay				
FoundationOne CDx, *n* (%)	194 (66.2%)	185 (66.1%)	9 (69.2%)	1.000
NCC Oncopanel, *n* (%)	60 (20.5%)	57 (20.4%)	3 (23.1%)	
FoundationOne liquid CDx, *n* (%)	39 (13.3%)	38 (13.6%)	1 (7.7%)	
Sampling method (excluding liquid biopsy)				
Biopsy, *n* (%)	194 (76.4%)	182 (74.6%)	12 (100.0%)	0.159
Surgery, *n* (%)	60 (23.6%)	60 (24.6%)	0 (0.0%)	
Sampling organ (excluding liquid biopsy)				
Pancreas, *n* (%)	131 (51.6%)	120 (49.6%)	11 (91.7%)	0.021
Liver, *n* (%)	88 (34.6%)	88 (36.4%)	0 (0.0%)	
Lymph node, *n* (%)	9 (3.5%)	9 (3.7%)	0 (0.0%)	
Other, *n* (%)	26 (10.2%)	25 (10.3%)	1 (8.3%)	

Abbreviations: CGP, comprehensive genomic profiling; TMB, tumor mutational burden.

**TABLE 2:** Rates of TMB‐high and MSI‐H in CGP assay.

Success rate of TMB measurement		Success rate of MSI measurement	
N/A, *n* (%)	—	N/A, *n* (%)	21 (7.2)
CBD, *n* (%)	18 (6.1)	CBD, *n* (%)	26 (8.9)
Measurable, *n* (%)	275 (93.9)	Measurable, *n* (%)	246 (84.0)

Result of TMB status		Result of MSI status	
TMB‐high, *n* (%)	13 (4.4)	MSI‐H, *n* (%)	4 (1.4)
TMB‐low, *n* (%)	262 (89.4)	MSS, *n* (%)	242 (82.6)

Abbreviations: CBD, cannot be determined; MSI, microsatellite instability; MSI‐H, microsatellite instability‐high; MSS, microsatellite stable; N/A, not available; TMB, tumor mutational burden.

### 3.2 Clinical and Genetic Characteristics of TMB‐High Cases

**FIGURE 2:**
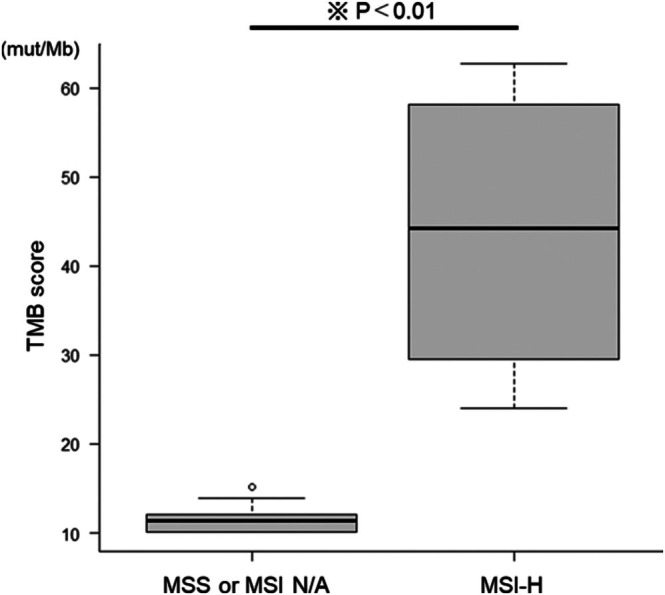
Comparison of tumor mutation burden (TMB) scores in patients with TMB‐high pancreatic cancer between the microsatellite stable (MSS) or microsatellite instability (MSI) unavailable group and the microsatellite instability‐high (MSI‐H) group.

**FIGURE 3:**
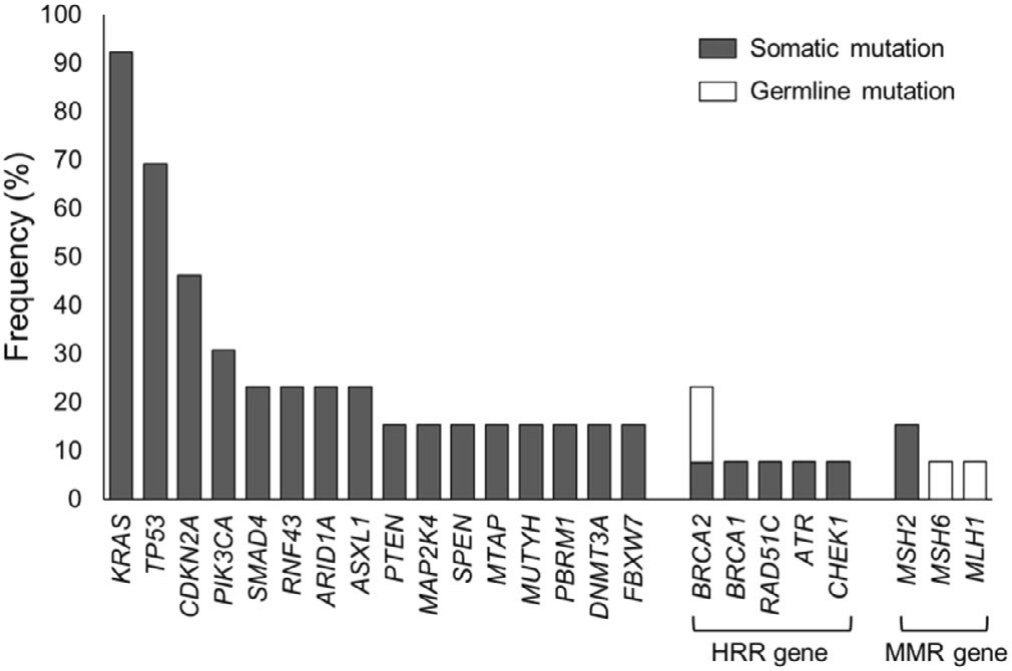
The frequencies of somatic and germline gene mutations in patients with tumor mutation burden (TMB)‐high pancreatic cancer.

Tables 3 and 4 show the clinical and genetic characteristics of 13 patients with TMB‐high pancreatic cancer. Of these, seven (53.9%) were males, with a median age of 71 years (range: 50–75 years). Histologic subtypes included adenocarcinoma (11 cases), Asc (one case), and ACC (one case). Four of the 13 patients had MSI‐H tumors, all of which were adenocarcinomas. The median TMB score was 11.38 mut/Mb (range: 10.09–15.13 mut/Mb) for the nine microsatellite stable (MSS)/MSI status unavailable cases and 44.25 mut/Mb (range: 23.96–62.76 mut/Mb) for the four MSI‐H cases, with a statistically significant difference between the two groups (*p* < 0.01) (Figure 2). Across all 13 cases, the median number of pathogenic variants detected via CGP was 6 (range: 1–27). Two patients (15.4%) had germline *BRCA2* mutations (known as the homologous recombination repair gene), both having MSS tumors. Of the two cases with germline *BRCA2* mutations, one was identified as germline by the NCC Oncopanel, and the other was initially tested with F1CDx but later confirmed to be germline by BRACAnalysis. In addition, one patient each (7.7%) had germline *MLH1* and *MSH6* mutations (known as DNA mismatch repair genes), and both cases had MSI‐H tumors. Mutations in the “big four” genes—*KRAS*, *TP53*, *CDKN2A*, and *SMAD4*—were frequently observed (Table 3). For example, *KRAS* mutations were detected in 12 of the 13 patients (92.3%). Figure 3 shows the overall genetic mutation frequencies, including those in other germline or somatic genes. Single‐occurrence somatic mutations are not included in Figure 3.

**TABLE 3:** Cases of patients with TMB‐high tumors.

Case	Age	Sex	Histology	CGP assay	MSI	TMB score (mut/Mb)	Number of pathogenic variants	Germline mutations	Somatic “big four” gene mutations				Pembrolizumab administration
1	73	Male	Adenoca.	NCC	N/A	11.60	1	—	*KRAS*	—	—	—	No
2	73	Female	Adenoca.	F1CDx	MSI‐H	23.96	5	—	*KRAS*	*TP53*	*CDKN2A*	—	Yes
3	62	Female	Adenoca.	F1CDx	MSS	10.09	7	—	*KRAS*	*TP53*	—	*SMAD4*	No
4	66	Female	Adenoca.	F1CDx	MSS	13.87	6	—	*KRAS*	*TP53*	*CDKN2A*	*SMAD4*	Yes
5	75	Male	Asc	F1CDx	MSS	10.09	7	—	*KRAS*	*TP53*	*CDKN2A*	—	No
6	75	Female	Adenoca.	F1CDx	MSS	15.13	6	—	*KRAS*	*TP53*	*CDKN2A*	—	No
7	70	Female	Adenoca.	F1CDx	MSS	10.09	9	—	*KRAS*	—	*CDKN2A*	—	No
8	74	Male	Adenoca.	NCC	MSI‐H	53.50	7	—	*KRAS*	*TP53*	—	—	Yes
9	71	Male	Adenoca.	NCC	MSS	10.10	2	*BRCA2*	*KRAS*	—	—	—	Yes
10	74	Male	Adenoca.	F1Liquid	MSS	11.38	5	—	*KRAS*	*TP53*	—	—	Yes
11	67	Female	Adenoca.	F1CDx	MSI‐H	62.76	27	*MLH1*	—	*TP53*	—	—	No
12	66	Male	Adenoca.	F1CDx	MSI‐H	35.00	11	*MSH6*	*KRAS*	*TP53*	*CDKN2A*	*SMAD4*	Yes
13	50	Male	ACC	F1CDx	MSS	12.07	4	*BRCA2*	*KRAS*	—	—	—	No

Abbreviations: ACC, acinar cell carcinoma; Adenoca., adenocarcinoma; Asc, adenosquamous carcinoma; MSI, microsatellite instability; MSI‐H, microsatellite instability‐high; MSS, microsatellite stable; TMB, tumor mutational burden.

**TABLE 4:** Personal and family cancer history in patients with TMB‐high tumors.

Case	Patient's history	Family history
1	No	Father: liver cancer
2	No	No
3	No	Father: oral cancer; Sibling: breast cancer
4	No	Mother: pancreatic cancer
5	No	Father: gastric cancer
6	No	Sibling: biliary tract cancer; Sibling: breast cancer
7	No	Child: breast cancer
8	No	Sibling: biliary tract cancer; Sibling: gastric cancer
9	Cutaneous squamous cell carcinoma	Father: prostate cancer
10	No	Father: gastric cancer
11	No	Sibling: colon cancer
12	No	Father: gastric cancer; Father: liver cancer; Mother: lung cancer
13	No	No

Abbreviation: TMB, tumor mutational burden.

### 3.3 Effects of Pembrolizumab in Patients With TMB‐High Pancreatic Cancer

**FIGURE 4:**
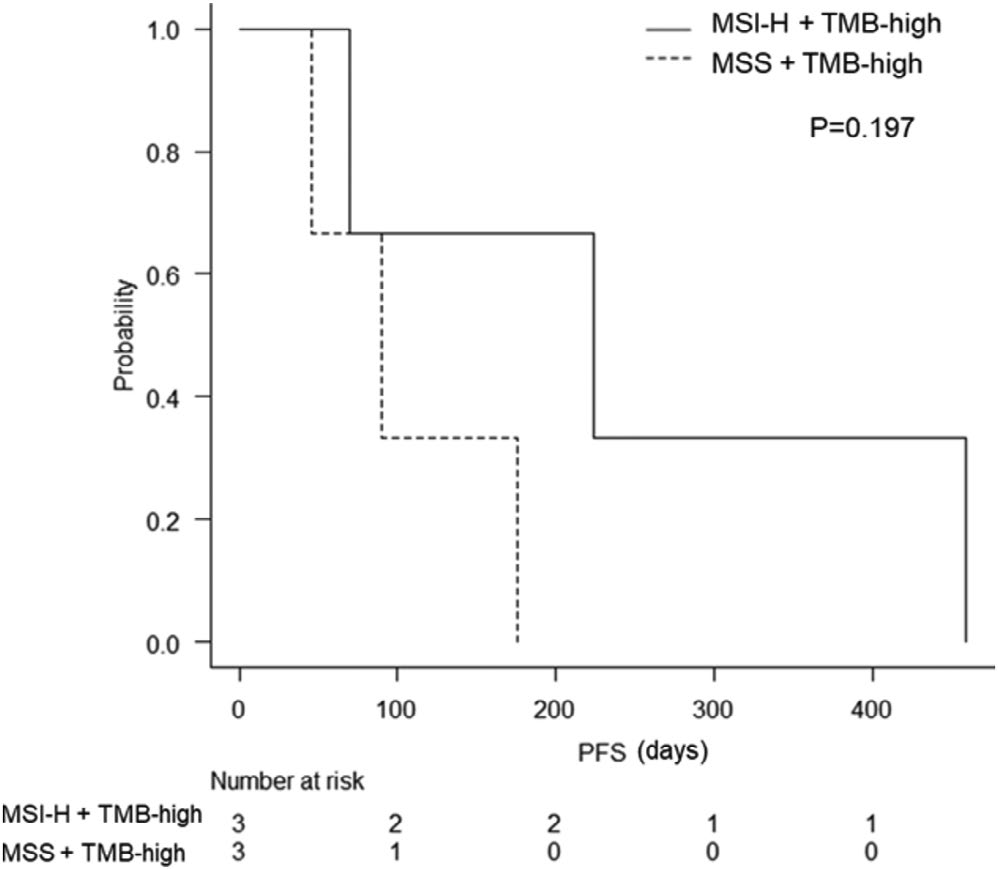
Progression‐free survival (PFS) according to pembrolizumab administration in patients with microsatellite instability‐high (MSI‐H) plus tumor mutation burden (TMB)‐high tumors and those with microsatellite stable (MSS) plus TMB‐high tumors by the Kaplan–Meier method.

Of the 13 patients in the TMB‐high group, six were administered pembrolizumab. Of the remaining seven, five were not administered pembrolizumab owing to lack of regulatory approval for TMB‐high at the time, one patient was lost to follow‐up with unknown subsequent treatment, and one continued their current regimen owing to sustained effectiveness. Table 5 lists the six patients treated with pembrolizumab. Four patients had a performance status (PS) of 0, and two had a PS of 1. Table 5 shows the number of previous treatment regimens. Three patients had tumors characterized as MSI‐H plus TMB‐high, and three had MSS plus TMB‐high. Among the six patients treated with pembrolizumab, two achieved a partial response (PR), two had stable disease (SD), and two had progressive disease (PD). This yielded an objective response rate of 33.3% and a disease control rate of 66.7%. The median number of pembrolizumab treatment cycles was 7 (range: 2–21), and the median PFS was 133 days (range: 46–459 days). In patients with MSI‐H plus TMB‐high, two of the three patients achieved PR. The median PFS for all three patients in this group was 227 days (range: 70–459 days). In contrast, no patients with MSS plus TMB‐high achieved a PR, whereas two of the three achieved SD. The median PFS for the three patients in this group was 90 days (range: 46–176 days). Figure 4 shows the Kaplan–Meier curves for PFS, comparing patients with MSI‐H plus TMB‐high and those with MSS plus TMB‐high administered Pembrolizumab. A trend toward longer PFS was observed in patients with MSI‐H plus TMB‐high, but the small size precluded a statistically significant difference (*p* = 0.197).

**TABLE 5:** Characteristics of patients with TMB‐high tumors treated with pembrolizumab.

Case	Age	Sex	MSI status	TMB score (mut/Mb)	PS	Number of previous regimens	Number of Pem courses	PFS	Best response^a^
2	73	F	MSI‐H	23.96	1	1	11	224	PR
4	66	F	MSS	13.87	0	3	9	176	SD
8	74	M	MSI‐H	53.50	0	1	3	70	PD
9	71	M	MSS	10.10	0	4	5	90	SD
10	74	M	MSS	11.38	1	4	2	46	PD
12	66	M	MSI‐H	35.00	0	5	21	459	PR

Abbreviations: F, female; M, male; MSI, microsatellite instability; MSI‐H, microsatellite instability‐high; MSS, microsatellite stable; PD, progressive disease; PFS, progression‐free survival; PR, partial response; PS, performance status; SD, stable disease; TMB, tumor mutational burden.

^a^ Evaluated based on the revised RECIST guidelines (version 1.1).

## 4 Discussion

In this study, 4.4% of patients with pancreatic cancer who underwent CGP exhibited TMB‐high. Previous studies show a TMB‐high frequency of 1%–2% among patients with pancreatic cancer, suggesting that the frequency observed in our cohort may be comparatively higher [20, 21]. When considering only the 275 patients for whom TMB could be reliably assessed, the TMB‐high rate increased slightly to 4.7%. Our analysis encompassed histological subtypes beyond adenocarcinoma, including Asc and ACC. However, even when restricting the analysis to adenocarcinoma cases alone, the TMB‐high frequency remained elevated at 4.0%. The demographic characteristics of our patient cohort—including sex, age, and the type of CGP assay used—were similar to those described in previous studies. Therefore, these variables are unlikely to explain the observed difference in TMB‐high frequency. However, our institution is a cancer center, and patients may have unique genetic backgrounds, such as a higher prevalence of multiple primary cancers or a family history of cancer compared with a general cohort. These factors may have contributed to the higher frequency of TMB‐high cases observed in our study.

When comparing patient backgrounds between the TMB‐low and TMB‐high groups, the TMB‐high group included a higher proportion of biopsy specimens than surgical specimens and more specimens obtained from primary pancreatic tumors. These findings are interesting; however, further studies with a larger sample size are needed to determine whether biopsy specimens from the pancreatic primary tumor via endoscopic ultrasound‐guided tissue acquisition are more likely to detect TMB‐high status than surgical pancreatic specimens and other types of samples.

Among the 13 TMB‐high cases, nine had TMB scores ranging from 10 to 20 mut/Mb. The relatively large proportion of cases with TMB values just above the threshold of 10 mut/Mb may have contributed to the elevated frequency of TMB‐high observed in this study. In this study, four of the 13 TMB‐high cases (30.8%) were identified as exhibiting MSI‐H. Notably, all MSI‐H cases also met the criteria for TMB‐high, with a median TMB score of 44.25 mut/Mb. A previous study shows that MSI‐H is a contributing factor to elevated TMB [23]. Our findings indicate that this association is also evident in pancreatic cancer.

In MSS plus TMB‐high cases, germline mutations in the *BRCA2* gene were identified in two patients. One of these patients had ACC, a histological type known to exhibit a high frequency of *BRCA1/2* mutations [24]. More proactive use of CGP in ACC may facilitate the identification of additional TMB‐high cases. Among the MSI‐H plus TMB‐high cases, germline mutations in the *MSH6* and *MLH1* genes—both associated with dMMR—were identified in one case each. In the TMB‐high cases analyzed in this study, mutations in the *KRAS*, *TP53*, *CDKN2A*, and *SMAD4* genes—commonly referred to as the “big four genes”—were observed at high frequencies. This is consistent with those typically found in PDAC cases [25, 26]. A study from the United States shows that the frequency of *KRAS* mutations is lower in TMB‐high cases [20]. However, neither the study conducted in Japan nor our current research revealed a significant difference in the frequency of *KRAS* mutations [21]. While racial differences may contribute to this phenomenon, further investigation is required to fully understand the underlying factors. In the TMB‐high cases analyzed in this study, the median number of pathological variants was six, which was higher than the previously reported median of four for pancreatic cancer [26].

Regarding the efficacy of pembrolizumab treatment, PR was observed exclusively in MSI‐H plus TMB‐high cases. However, SD was observed in two of the three MSS plus TMB‐high cases, suggesting that pembrolizumab may also offer some therapeutic benefit in this subgroup. Regarding PFS, the median duration was 227 days in MSI‐H plus TMB‐high cases, compared to 90 days in MSS plus TMB‐high cases, the latter being shorter than the former. In the MSS plus TMB‐high group, the efficacy of pembrolizumab monotherapy appears to be limited, highlighting the need to develop more effective treatment strategies. Moreover, the universal applicability of the TMB‐high threshold of 10 has been questioned [27, 28]. In this study, Case 8, who had a high TMB score (> 50), experienced disease progression, while others with relatively lower TMB scores (10–20)—such as Cases 4, 9, and 10—achieved SD. These findings suggest that additional biomarkers, either independently or in combination with the TMB score, may be necessary to more accurately predict the efficacy of pembrolizumab.

This study had some limitations. First, the small number of TMB‐high cases limited our ability to thoroughly investigate genetic characteristics and assess the efficacy of pembrolizumab. Therefore, further research is warranted, including studies from other institutions and those involving larger sample sizes. Second, owing to the retrospective nature of the study, the number of prior treatment regimens and patient clinical conditions at the initiation of pembrolizumab therapy varied. Furthermore, as this study focused on patients with TMB‐high pancreatic cancer, comparisons of prognosis or treatment response between patients in the TMB‐high and TMB‐low groups could not be performed. Given the rarity of TMB‐high pancreatic cancer, a multicenter prospective study evaluating pembrolizumab treatment is warranted.

## 5 Conclusions

The frequency of TMB‐high status in advanced pancreatic cancer was approximately 4.4%, which is slightly higher than previously reported findings. As observed in other solid cancers, identifying TMB‐high cases through CGP may provide therapeutic benefits from pembrolizumab treatment. While some therapeutic efficacy of pembrolizumab was observed in patients with MSS plus TMB‐high pancreatic cancer, the clinical response appeared more pronounced in patients with MSI‐H plus TMB‐high pancreatic cancer. These findings highlight the need for further development of more effective therapeutic agents and treatment strategies, especially for patients with MSS plus TMB‐high pancreatic cancer.

## Author Contributions

Conceptualization, data curation, formal analysis, investigation, methodology, project administration, resources, visualization, and writing – original draft: Y.K. and K.I. Investigation and writing – review and editing: K.K., M.U., R.T., T.K., T.M., M.K., H.K., K.H., K.M., T.N., and N.S. Data collection, data curation, and writing – review and editing: Y.Y. Investigation, supervision, and writing – review and editing: K.O.

## Funding

The authors have nothing to report.

## Ethics Statement

This study was approved by the Institutional Review Board of Osaka International Cancer Institute (Approval No. 20148‐8) and was designed and conducted in accordance with the ethical standards of the 1964 Declaration of Helsinki and its subsequent amendments.

## Conflicts of Interest

Y.K. received honoraria for lectures from Incyte Biosciences Japan. K.I. received honoraria for lectures from Taiho Pharmaceutical, Incyte Biosciences Japan, AstraZeneca, MSD, Nihon Servier, Chugai Pharmaceutical, Guardant Health Japan, and Myriad Genetics, and research funding from ASKA Pharmaceutical. R.T. received honoraria for lectures from Taiho Pharmaceutical, Hisamitsu Pharmaceutical, Novartis, MSD, Myriad Genetics, and TEIJIN PHARMA. N.S. received honoraria for lectures from Chugai Pharmaceutical, Daiichi Sankyo, and MSD. K.O. received honoraria for lectures from Chugai Pharmaceutical, Eisai, AstraZeneca, Incyte Biosciences Japan, and Nihon Servier, and research grants from Sumitomo Chemical. The other authors declare no conflicts of interest.

## Data Availability

The data that support the findings of this study are available from the corresponding author upon reasonable request.
